# Stigma reduction in relation to HIV test uptake in low- and middle-income countries: a realist review

**DOI:** 10.1186/s12889-018-6156-4

**Published:** 2018-11-20

**Authors:** Subash Thapa, Karin Hannes, Margaret Cargo, Anne Buve, Sanne Peters, Stephanie Dauphin, Catharina Mathei

**Affiliations:** 1 Research Unit of General Practice, Department of Public Health, University of Southern Denmark, Odense, 5000 Denmark; 2 Social Research Methodology Group, Faculty of Social Sciences, KU Leuven, Parkstraat 45, 3000 Leuven, Belgium; 3 Health Research Institute, University of Canberra, University Drive, 22-B17, Bruce, ACT 2601 Australia; 4 Department of Public Health, Institute of Tropical Medicine, Nationalestraat 155, 2000 Antwerp, Belgium; 5 Department of Public Health and Primary care, KU Leuven, Kapucijnenvoer 33, 3000 Leuven, Belgium

**Keywords:** Context-mechanism-outcome configurations, HIV test uptake, Low- and middle-income countries, Realist review, And stigma reduction interventions

## Abstract

**Background:**

This realist review was conducted to understand how stigma is reduced in relation to HIV test uptake in low- and middle-income countries (LMICs).

**Methods:**

A systematic search of eight databases resulted in 34 articles considered for synthesis. Data synthesis was guided by a preliminary programme theory and included coding the meaning units to develop themes or intervention pathways that corresponded to context-mechanism-outcome configurations.

**Results:**

We found that the interventions produced an effect through two pathways: (a) knowledge leads to changes in stigmatizing attitudes and increases in HIV test uptake and (b) knowledge and attitudes lead to changes in stigmatizing behaviours and lead to HIV test uptake. We also found one competing pathway that illustrated the direct impact of knowledge on HIV test uptake without changing stigmatizing attitudes and behaviour. The identified pathways were found to be influenced by some structural factors (e.g., anti-homosexuality laws, country-specific HIV testing programmes and policies), community factors (e.g., traditional beliefs and practices, sexual taboos and prevalence of intimate partner violence) and target-population characteristics (e.g., age, income and urban-rural residence).

**Conclusions:**

The pathways and underlying mechanisms support the adaptation of intervention strategies in terms of social context and the target population in LMICs.

## Background

According to the United Nations Programme on HIV/AIDS (UNAIDS), there are approximately 37 million people worldwide living with HIV and almost 95% of people living with HIV (PLWH) reside in low- and middle-income countries [1]. Sub-Saharan Africa is the most affected region, with an estimated 26 million people living with HIV. An estimated 40% of people globally still need to access HIV testing service to know their HIV status, and the vast majority of them are in LMICs [2]. Where available, one of the reasons for the lower uptake of the HIV testing service in most LMICs is HIV stigma [3].

The Joint United Nations Programme on HIV/AIDS defined HIV stigma as a process of devaluation of people either living with or associated with HIV infection [4]. Stigma is both the behaviours of people without the disease (e.g., labelling, denial, exclusion) that leads to the devaluation of the people living or associated with HIV (public-stigma) and negative feelings of being devalued (e.g., fear, shame) among the people with the disease (self-stigma) [5]. PLWH have been stigmatized because the disease is generally perceived as dangerous, contagious, and associated with behaviours outside of social norms [6]. HIV stigma may have serious consequences, such as loss of friendship and family ties, dismissal from school and occupation, and denial of health care [7]. HIV stigma is associated with lower uptake of HIV testing services, non-disclosure and delayed entry into comprehensive health care, which further lead to higher transmission rates [3].

Several interventions to reduce HIV stigma have been developed and evaluated for their impacts on HIV test uptake. Some interventions have context-specific effects [8]. For example, interventions to increase access to HIV testing through a home-based testing approach in Kenya [9] and Zambia [10] were noted to reduce HIV stigma and increase HIV test uptake. However, an intervention based on mass media and interpersonal communication strategies in South Africa (2007) reported having some effect on HIV knowledge but limited effects on stigmatizing attitudes and HIV testing uptake [11]. An intervention, or set of interventions, may succeed or fail depending on the wider social systems in which they are implemented [12].

Stigma reduction interventions are complex. These interventions have multiple components: partly individual-related and partly resource-related [13]. The interaction between elements related to individuals and their sociocultural environment is a significant reason why HIV interventions succeed or fail [12]. For instance, individuals do not always make the same choices about their behaviours in a similar pattern every time. However, some choices (e.g., HIV testing) might be more likely than others depending on the opportunities and resources provided by the interventions.

Although individual behaviours and choices cannot be fully predicted, public health researchers can identify non-linear semi-predictable patterns of choice-making that occur under certain circumstances following an intervention. There is evidence showing that interventions have been effective in reducing stigma and increasing HIV test uptake; however relatively little is known on how, why, for whom and in which circumstances particular stigma reduction interventions work [9, 10] .

### Why conduct this review?

This review aims to address whether and how HIV stigma can be reduced to maximize public health benefits via increasing HIV test uptake in LMICs. Especially in LMICs, low income along with illiteracy and traditional beliefs may influence the association between stigma reduction and HIV test uptake [8]. Moreover, there is heterogeneity in the ways in which HIV stigma is experienced across different communities, and it is likely that the interventions and mechanisms that work to reduce HIV stigma and increase HIV testing may also vary between communities and individuals. The potential different pathways leading people to or pulling them away from HIV testing, thus, need to be unpacked [8]. Therefore, we conducted this realist review to understand how stigma is reduced in relation to HIV test uptake in LMICs.

The aim was to develop programme theory to uncover the mechanisms operating in stigma reduction interventions to increase HIV test uptake in particular contexts. A programme theory provides plausible explanations of why certain interventions work or do not work in certain circumstances [14]. Consequently, the underlying mechanisms would guide programme managers to design better public health interventions and understand which stigma reduction interventions should be implemented for whom and in which contexts.

## Methods

A realist perspective was chosen because it allows for the evaluation of complex social interventions [15]. The realist perspective is a theory-driven and multi-method-based research methodology that uses an interpretive approach to synthesize evidence to reveal how intervention strategies interact with context to trigger mechanisms and produce outcomes. In a realist review, first, a preliminary programme theory is developed that explains how context influences mechanisms to generate outcomes. The preliminary theory is represented as a set of context-mechanism-outcome (CMO) configurations [15]. Then, the preliminary programme theory is iteratively refined based on a systematic review of empirical evidence to investigate whether, why or how intervention strategies produce observed outcomes, for whom and in what circumstances. The refined programme theory is a middle range theory that is useful for decision makers to identify “What works, for whom, in what respects, to what extent, in what contexts, and how?”

A middle range theory is understood as “theory that lies between the minor but necessary working hypotheses … and the all-inclusive systematic efforts to develop a unified theory that will explain all the observed uniformities of social behaviour, social organization and social change” [16]. Pawson and Tilley argued that middle range theories are specific enough to generate particular propositions to test and are general enough to apply across different situations [12]. At best, a realist review can indicate the conditions in which the intervention works (or not) and how it does so, which allows programme managers and decision makers to assess whether interventions that proved effective in one setting may do so in another setting and assists programme planners in adapting interventions to suit specific contexts.

### Preliminary Programme theory

**Fig. 1:**
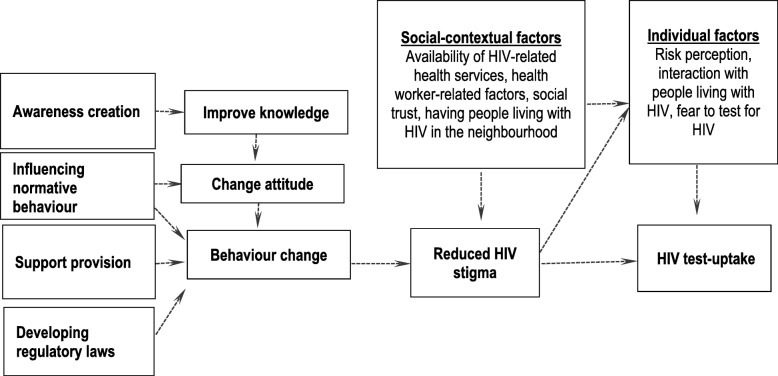
Preliminary programme theory explaining the effect of stigma-reduction intervention strategies on HIV test uptake

This realist review was informed by a scoping review of empirical and grey literature on stigma reduction interventions to develop a preliminary programme theory (see Figure 1), which has been published elsewhere [8]. The scoping review resulted in an initial programme theory consisting of 4 types of intervention strategies (i.e., awareness creation, influencing normative behaviour, providing support, and developing regulatory laws), three proximal outcomes (i.e., improvement of knowledge, change of attitude and behaviour) and one distal outcome (i.e., HIV test uptake). The preliminary programme theory conceptualizes HIV stigma as an overarching term that contains three elements: problems of knowledge (ignorance), problems of attitudes (prejudice), and problems of behaviour (discrimination) [17].

The awareness creation strategy generally improves knowledge about HIV and people diagnosed with HIV, and the influencing normative behaviour strategy changes stigmatizing attitudes and behaviours, and subsequently, increases HIV test uptake. Intervention strategies providing support to the people living or associated with HIV, and developing regulatory law change the stigmatizing behaviours of the people, and subsequently, increase HIV test uptake. The framework further outlines that the mechanisms described are influenced by the interaction of various social-contextual and individual factors. Based on the initial scoping review, the following two research questions guided the review: (1) Does stigma reduction lead to HIV test uptake? (2) What are the pathways leading to HIV test uptake?

### Search strategy

To bring together different sources of evidence that support, refine, or refute our preliminary programme theory, we performed a systematic search in the following eight electronic databases: PubMed, Excerpta Medica Database (EMBASE), POPLINE, PsycINFO, Sociological Abstracts, Web of Science, Scopus and the Cumulative Index to Nursing and Allied Health Literature (CINAHL). We also performed an opportunistic purposeful searching process in ‘Google scholar’, 3ie database, trail registers from Campbell International Development Coordinating Group and the databases of two international organizations, namely the World Health Organization (WHO) and UNAIDS to enhance the possibility of identifying ‘grey’ literature [18]. Opportunities were sought to find additional studies from the reference list of primary studies.

### Screening and study selection

We screened the articles in two phases. In the first phase, we included the articles based on the following core inclusion criteria: (a) dealt with HIV-related interventions that addressed causes of HIV stigma or included intervention components to reduce HIV stigma; (b) based in the low- or middle-income countries; (c) quantitative, qualitative or mixed-method studies, or programme reports and policy documents that described various forms of stigma or compared CMO configurations between different stigma reduction intervention strategies; (d) written in English; and (e) the outcome was related to HIV test uptake or for non-quantitative studies, the impact of stigma on HIV-test uptake was featured. The papers that were published until the year 2016 were considered for inclusion. Two independent reviewers screened the titles, abstracts and keywords of the identified documents.

In the second phase, the abstracts from the first phase were checked specifically against any one of the following three criteria: (a) whether the abstract described the association between stigma reduction and HIV test uptake, (b) whether the abstract referred to behaviour change relating to stigma reduction and HIV test uptake, and (c) whether the abstract described the social contextual factors influencing stigma reduction and its association with HIV test uptake.

Abstracts were coded as ‘Yes’ if any of the three-inclusion criteria were satisfied, and ‘No’ if none of the criteria were met. After the screening of abstracts for the second time, the full-text of articles were retrieved and evaluated by two independent reviewers to ensure that one or more of our inclusion criteria were met. Disagreements about articles to be included and excluded were resolved through consensus between the reviewers. Documents were included in the review based on relevance, that is, the extent to which they informed the development of the theoretical framework or clarified the CMO configurations [19].

### Data extraction

For each included article, data were extracted independently by two researchers based on a data extraction tool. The contents of the preliminary programme theory were embedded in the data extraction form, and this provided a template to interrogate ‘what works, for whom, in what circumstances’. To test the usability and functionality of the data extraction form, the tool was pretested on three purposefully selected articles [20]. The evidence table was designed to summarize the author/date, study aim, intervention, intervention strategies, study area, study type, population, outcomes, mechanisms involved, effectiveness and contextual factors for all the studies included. Direct quotations from qualitative studies were considered very informative and were accompanied by the name of the author and the year of the publication. The researchers compared and discussed their findings, and tried to reach consensus on the most important evidence presented in each article.

### Quality appraisal

To assess the quality of primary studies, the Mixed Methods Appraisal Tool (MMAT)-Version 2011 (Department of Family Medicine, McGill University, Canada) was used. MMAT is one of the few tools that appraises the quality of diverse study designs [21] and provides a score that refers to the number of criteria met divided by four to calculate the percentage of the study quality for both quantitative and qualitative studies. For a mixed-methods study, the overall quality score is the lowest percentage of either the qualitative method or the quantitative method. We considered the score of 75% or above as ‘high quality’. We did not make decisions to exclude any of the studies based on the study quality. We believed that the information about the study quality would complement the synthesis process by informing whether a particular inference drawn from a primary study was based on sufficient evidence to make a methodologically credible contribution to the theoretical framework [14]. Data from one study can be used to make sense of a pattern in another, and other sources may be used to build explanations [14].

### Data synthesis

In this review, all the quantitative and qualitative data were analysed narratively. First, the data extracted from each study using the data extraction tool were summarized and organized in one evidence table (see Table 1). Extracted data from each primary study were coded for meaning units relevant to context, strategies, and mechanisms related to HIV test uptake. Next, themes were developed from the initial codes that corresponded to the preliminary theory. We then grouped several mechanisms, such as changed fear, changed shame, and increased tolerance of PLWH, according to the theme of attitudinal mechanisms (see Table 2). A focal point of the analysis was using the attitudinal mechanisms as a basis for constructing the context-mechanism-outcome pathways. For example, we identified the pathways as ‘knowledge leading to changes in stigmatizing attitudes and increases in HIV test uptake’ from assessing the relationships between the themes and considering the outcomes reported in the primary studies. The relationships between the themes were generated based on how the information was reported in the primary studies.

**Table 1:** Summary of included studies

Author/date	Study aim	Intervention	Intervention strategies	Country	Study type	Population	Outcome	Mechanisms	Effectiveness	Contextual factors
Apinundecha, 2007	To investigate the effect of the intervention to reduce stigma	Socio-economic support for community participation in HIV prevention	Awareness raising, community mobilization and support provision	Thailand	RCT	General population	Changes in knowledge and attitude	Improved knowledge, reduced fear, increased interaction with PLWH (Pathway 2)	The interventions reduced resource constraints by empowering the community, and providing financial support and this was an effective means of increasing interaction between PLWH and other community members, increasing tolerance and reducing HIV/AIDS stigma. The effectiveness in terms of HIV testing uptake was not reported.	
Balfour, 2013	To compare HIV knowledge, stigma and health care seeking behaviours	“On The Ball” program: pictures, question and answers, key statements, group activities, and soccer coaching	Raising awareness	South Africa	RCT	School children	Changes in Knowledge, attitude	Improved knowledge, change attitude	Elementary students who participated in the program reported greater HIV knowledge and lower HIV stigma (*p* < .001) than those who had not; but the effectiveness in terms of HIV test uptake was not reported	
Berendes, 2011	To test that knowledge and self-efficacy would serve as facilitators for testing	Mass-media program and community level activities	Awareness raising	Malawi	Cross sectional	General population	Higher levels of knowledge and intentions to test	Improved knowledge, changed attitude and increased self-efficacy for testing (Pathway 1)	Effectiveness was not reported in terms of HIV test uptake. A positive association was found between program exposure, and knowledge, low levels of stigma, increased self-efficacy and intentions to test.	Younger age and being educated were more likely to be tested
Blas, 2013	To develop culturally-appropriate messages to motivate MSM to get tested for HIV	Internet and mobile phone-based messaging	Raising awareness	Peru	Qualitative: Focus groups	MSM	Change in knowledge, and reduced fear	Improved knowledge and reduced fear	Effectiveness was not reported in terms of HIV test uptake but provided information on how to make educational message appropriate to overcome fear of testing	
Castro, 2005	To assess the relationships between stigma and integrated HIV prevention and care	ART access, VCT access, health education and involvement of PLWH in health care	Raising awareness, health service provision, and community mobilization	Haiti	Literature review with a descriptive case study	General population	HIV knowledge and attitude, HIV test uptake	Improved knowledge, changed attitude (Pathway 1)	The introduction of quality HIV care can lead to a rapid reduction in stigma, with resulting increased uptake of testing.	Improving quality of health care and increasing health services access increases staff morale, reduce work place stigma and increase HIV test uptake
Chung, 2015	To determine whether knowledge about HIV and self-efficacy associated with stigma			Namibia	Cross-sectional	General population	Stigma reduction	Improved knowledge, changed attitude and increased self-efficacy for testing	Effectiveness was not reported in terms of HIV test uptake.	Stigma tended to decrease with age and years of education
Coates, 2014	To test community-based VCT would be effective	Community testing, post-test support services, community mobilization via social networks and information sessions	Raising awareness, health service provision, community mobilization, and support provision	Thailand, South Africa, Tanzania and Zimbabwe	RCT	General population	Change in attitudes and HIV test uptake	Improved knowledge, changed community norms (Pathway 1)	Community-based VCT increased testing rates by 25% overall (12–39; *p* = 0.0003), by 45% (25–69; *p* < 0·0001) in men and 15% (3–28; *p* = 0.013) in women. Social norms regarding HIV testing were improved by 6% (95% CI 3–9) in communities in the intervention group.	Logistical barriers influenced the effect
Colchero, 2016	To estimate the impact of the behavioural, biomedical, and structural interventions across a range of outcomes	prevention kits, educational messages, peer-led program, interview and workshops	Raising awareness, and health service provision	Mexico	Quasi-experiment	MSM, transgender, male sex workers, health workers, police officers	Change in knowledge, attitude, behaviour and HIV test uptake	Improved knowledge, reduced stigma, changed behaviour and increased HIV test uptake (Pathway 2)	Per additional year of program exposure, there was a 7% reduction in stigma/discrimination from healthcare personnel relative to baseline coverage; a 7.5% increase in HIV testing; a 6.3% increase in awareness of HIV status among HIV-positive individuals a 6.7% increase in HIV-positive individuals on treatment.	
Derksen, 2014	Reduce stigma between potential sexual partners and increase HIV testing rates by providing new information about the effect of ART on HIV transmission risk	One health information community meeting in each village about benefits of ART access, ART provision	Raising awareness and health service provision	Malawi	RCT (Poster)	General population	Change in knowledge, attitude and HIV test uptake	Improved knowledge, changed attitude and increased HIV test uptake (Pathway 1)	Due to increased ART access, the intervention was reported to increase knowledge, reduce fear and increase HIV test uptake	
Doherty, 2013	To compare the effect of home based vs. facility based HIV testing	HBCT mobilizing local counsellors for community mobilization and discussions	Raising awareness, Health service provision, and community mobilization	South Africa	RCT	General population	Change in Knowledge and attitude, stigmatizing behaviours, HIV testing	Improved knowledge, changed behaviours (Pathway 2)	69% of participants in the home based HCT arm versus 47% in the control arm were tested for HIV (prevalence ratio 1.54, 95% confidence interval 1.32 to 1.81). Participants in the intervention arm were less likely to report stigmatising behaviours.	Intimate partner violence was reduced
Hutchinson, 2007	To examine the effect of intervention in HIV knowledge and attitude, condoms use and HIV disclosure	Mass media and interpersonal communication	Raising awareness	South Africa	Cross-sectional	General population	Knowledge, HIV stigmatizing attitude, HIV testing and disclosure	Improved knowledge	The intervention was not reported to reduce stigmatizing attitude and HIV testing; however, mass media exposure increased the likelihood of talking to someone about HIV	Improvements in the quality and availability of HIV services at the local clinic also influenced the effect on stigma reduction
Jurgenson, 2013	To investigate whether home-based Voluntary Counselling and Testing has an impact on stigma	HBCT by lay counsellors, community mobilization, radio program	Raising awareness, health service provision, and community mobilization	Zambia	RCT	General population	Change in knowledge, attitude and HIV test uptake	Improved knowledge and changed attitude (Pathway 1)	7% reduction in stigma from baseline to follow-up, due to a reduction in individual stigmatizing attitudes. Being tested for HIV was associated with a reduction in stigma (beta = −0.57, *p* = 0.030), and there was a trend towards home-based VCT having a larger impact on stigma than other testing approaches (beta = − 0.78, *p* = 0.080 vs. beta = − 0.37, *p* = 0.551), possibly explained by a strong focus on counselling and the safe environment of the home.	
Jurgensen, 2013 (the 7 C’s)	To investigate the feasibility and acceptance of home-based VCT	HBCT by local counsellors;	Raising awareness, health service provision, and community mobilization	Zambia	Mixed-methods	General population	Change in knowledge, attitude and HIV test uptake	Improved knowledge, changed attitude and change behaviour (Pathway 1)	Social mobilisation lead to significant reduction in stigma (*P* < 0.001) in both the intervention and control arms.	Local counsellors ensured community trust in the services
Lapinski, 2008	To assess the effects of the film about an HIV positive man	Film (educational entertainment approach)	Raising awareness	Nigeria	Quasi-experimental	General population	Increase knowledge and intentions to test	Increased knowledge and intentions to test (Pathway 3)	The intervention changed male participants’ fear of the severity of HIV, less blame to PLWH and intentions to test. Women had negative attitude toward HIV following the intervention	
Low, 2013	To assess the effect of HBCT and community leader mobilization on HIV stigma	HBCT: community sensitization program	Raising awareness, health service provision, and community mobilization	Kenya	RCT	General population	Change in attitude and HIV testing	Improved knowledge, changed attitude and changed behaviour (Pathway 2; and Pathway 3)	Due to its “whole community” approach.,the home-based HIV testing intervention resulted in community leaders reporting lower levels of stigma; however, stigma among community members reacted in mixed ways	Bringing HIV testing closer to an individual reduced social-cultural barrier.
Ma, 2008	To compare the attitudes and acceptance of VCT and levels of HIV knowledge	ART, health care and education, PMTCT, VCT	Raising awareness, and health service provision	China	Cross-sectional	General population	Attitude, acceptance of VCT	Improved knowledge (Pathway 3)	Urban residents of program area had higher HIV/AIDS knowledge levels than urban residents of the comparison area (*p* = 0.002) and no significant differences in uptake were found between intervention and control areas	Higher education levels and income influenced the association
Mall, 2013	To assess changes in stigma, knowledge and VCT over time	HIV awareness and education campaign and access to ART	Raising awareness, and health service provision	South Africa	Cross-sectional	General population	Change in knowledge, HIV testing uptake	Improved knowledge, changed attitude and increased HIV test uptake (Pathway 1)	Overall basic knowledge of HIV/AIDS increased from 2004 to 2008 (*p* = 0.04) and stigmatisation towards HIV-positive individuals decreased over the same period (p < 0.001); and the proportion of participants who had undergone HIV testing increased from 2004 to 2008 (40 vs. 70%, respectively) and VCT increased from 26 to 43%.	Knowing someone infected, being female and being educated were associated with lower stigma levels
Maman, 2014	To assess attitudinal and behavioural changes in HIV testing norms, discussions, and stigma	Community Mobilization, Increased Access to VCT, Post-Test Support Services	Raising awareness, health service provision, community mobilization, and support provision	Tanzania, Zimbabwe, South Africa and Thailand	Qualitative: in-depth interviews	General population	Increase testing and change in attitude and behaviour	Improved knowledge changed community norms and changed behaviour and HIV test uptake (Pathway 2)	A change in HIV-related stigma over time was most pronounced in Tanzania and Zimbabwe. Participants in the intervention communities from these two sites attributed community-level changes in attitudes.	
Massey, 2012	To assess the effectiveness of the intervention to facilitate knowledge, attitudinal and behavioural change	Peer-led, school-based clubs based raising awareness (Songs, articles, dialogues and other media was used)	Raising awareness, and community mobilization	Senegal	Quasi-experimental	School children	Positive attitudes and intentions related to HIV test-uptake	Improved knowledge and attitude (Pathway 1)	Students exposed to intervention activities had 1.5 greater odds of intending to get HIV tested compared with students not exposed to the program.	Gender norms in sub-Saharan Africa reinforced and supported higher rates of HIV testing among women
Maughan, 2014	To examine the independent effects HIV- stigma on HIV testing			South Africa	Cross-sectional	General population	Stigma, HIV test uptake	Improved knowledge and reduced fear	Effectiveness was not reported in terms of HIV test uptake	
Moshabela, 2016	To understand the social, economic and contextual factors that affect Treatment as prevention program	‘Test and treat program’ and mobilization of local counsellors (traditional healers)	Raising awareness, health service provision, and community mobilization	South Africa	Qualitative: focus groups	PLWH, general population	HIV testing	Improved knowledge, changed attitude, changed behaviour and HIV test uptake (Pathway 2)	Traditional practitioners were engaged with the home-based testing services and HIV clinics; and specifically, home-based testing services were perceived as relatively successful in increasing access to HIV testing.	Witchcraft beliefs and illiteracy
Mukulo, 2013	To assess relationship of negative labeling and social exclusion in and attitudes toward VCT			Mozambique	Cross-sectional	General Women	Attitude towards HIV testing	Improved knowledge and changed attitude and behaviour	A decrease from 50 to 25-points in the score for social exclusion stigma was associated with 1.5 and 1.3-fold increase in odds for past-6-months VCT use and supporting VCT use	Contact with traditional healers were each associated with higher odds of supporting VCT
Murray, 2010	To assess access to VCT among MSM and transgender	VCT (rapid test: mobile units)	Raising awareness, and health service provision	Brazil	Commentary	MSM and transgender	HIV testing	Improved knowledge and changed attitude	Did not report effectiveness of interventions	Partnership between NGOs and public health services was crucial
Pappas, 2008	To examine associations between exposure to serial drama and outcomes related to HIV testing	Radio-based awareness programs, community meetings, messages in local magazine	Raising awareness	Botswana	Cross sectional study	General population	Knowledge and intentions to test	Improved knowledge, changed attitude, intention to test (Pathway 1)	Positive associations was found between exposure to the program and intermediate outcomes, including lower level of stigmatizing attitudes, stronger intention to have HIV testing, and talking to a partner about testing.	Increased access to HIV testing via national VCT program
Pulerwitz, 2015	To evaluate the relative effectiveness of interventions in reducing stigma	Quality of care policy, staff training, material supplies	Raising awareness, support provision, and regulatory law	Vietnam	Quasi experimental	Health care workers	Knowledge and attitude	Improved knowledge and reduced fear	Effectiveness was not reported in terms of HIV test uptake but, stigma measures had improved significantly for both intervention groups	The Law of HIV/AIDS Prevention and Control, which made HIV-related stigma an offence, and promoted full rights to PLWH
Raoura, 2008	To investigate the interplay between ART scale-up, different types of stigma and VCT uptake	ART and VCT	Raising awareness, and health service provision	Tanzania	Qualitative study: in depth interviews	community leaders, ART clients and health care providers	Knowledge and attitude	Reduced fear and internalized stigma and increased blame and increased HIV test uptake (Pathway 1)	The intervention reported a substantial increase in VCT uptake due to normalization of HIV but it also increased blaming attitude that can reduce VCT uptake	Beliefs on witchcraft
Semugoma, 2012	To investigate the potential health effects of the proposed anti homosexuality law among MSM			Uganda	Commentary	MSM and transgender	Fear of testing		Effectiveness was not reported in terms of HIV test uptake	Anti-homosexuality law and mandatory reporting of sexual identity and HIV positive test results by health workers increased fear and stigma
Uys, 2009	To assess the impact of stigma reduction intervention among the nurse and PLWH	Workshop: (1) sharing information, (2) increasing contact with the affected group, and (3) improving coping through empowerment	Raising awareness, and support provision	Lesotho, Malawi, South Africa, Swaziland, and Tanzania	Mixed methods: Multiple-case study design	A group of PLWH and nurses	Change in attitude and behaviour, HIV testing uptake	Reduced stigma among PLWH, but not among nurses; increased mutual support between nurses and PLWH (Pathway 3)	No change in stigma was reported among nurses but a significantly higher percentage of the nurse were tested for HIV; stigma experience of PLWH can be decreased, but that the stigma experiences of nurses are less easy to change	
van Royaan, 2016	To assess the impact of intervention on HIV testing, disclosure, stigma and discrimination	Family-based counselling and testing, behavioural intervention, mobilization of community local counsellors, training	Raising awareness, health service provision, and community mobilization	South Africa	Qualitative: In-depth interviews, focus groups	General population	Changes in Knowledge, attitude, HIV testing	Improved knowledge and changed attitude, and increased HIV test uptake (Pathway 1)	The family-based intervention encouraged HIV testing of adults, children, and adolescents and disclosure of HIV status. Intergenerational communication was identified as the key causal pathway to improve testing, linkage to care, disclosure, and reduced stigma for this group.	Hierarchical relationships between generations, inability to discuss sex across generations, and poor communication skills and sex as a taboo
Weihs, 2014	To provide a better understanding of employees´ experiences of a VCT	Lottery incentive scheme for testing	Awareness raising, and support provision	South Africa	Quasi-experimental study	Staffs General population	Increased knowledge attitude and HIV test uptake	Improved knowledge, reduced fear, reduced work place discrimination and increased HIV test uptake (Pathway 2)	Lottery induced excitement facilitated social interactions pertaining to HCT that mitigated the burden of HIV stigma in the workplace and created open discussions.	
Weiser, 2006	To assess knowledge and attitude toward testing, and prevalence and correlates of testing	Radio TV messages, and routine testing	Raising awareness, and health service provision	Botswana	Cross-sectional	General population	Change in Knowledge, attitude, HIV test uptake	Improved knowledge	Effectiveness was not reported in terms of HIV test uptake; routine testing appears to be widely supported and may reduce barriers to testing	
White, 2013	To qualitatively assess service provider and user attitudes of the quality of the various services	Integrating HIV and RH services, and community mobilization	Raising awareness, and health service provision	Cambodia	Qualitative: In-depth Interviews	Pregnant women	Stigma reduction, HIV testing	Increased knowledge and attitude, c hanged behaviour and HIV test uptake (Pathway 2)	Success stories of home based counselling team and integrated approach may reduce stigma and increase HIV testing, increase closeness to HIV testing	Increased visibility of HIV and family support influenced HIV test uptake
Wu, 2008	To reduce stigma and increase level of comfort working with PLWH for service providers in China	Mass media and a community advisory board involving PLWH and local people	Raising awareness	China	RCT	Health service providers	Change in Knowledge and fear	Improved knowledge and reduced fear among health care workers	Effectiveness was not reported in terms of HIV test uptake, but the intervention was successful to reduce stigma and discrimination among health care workers	Mandatory reporting of positive HIV test
Young, 2010	To determine the efficacy of community-based voluntary counselling and testing	Community-based HIV mobile voluntary counselling and testing, community mobilization, and post-test support services	Raising awareness, health service provision, community mobilization, and support provision	South Africa	Cross-sectional analysis of data from a RCT	General population	Change in Knowledge, attitude and HIV testing	Improved knowledge, changed community norms related to HIV, and enhance social support	Effectiveness was not reported in terms of HIV test uptake; however previous testing was found to be effective to reduce HIV stigma	Older generation, females and more educated people were more likely to have been tested.

Note. *ART* Antiretroviral Therapy; *HBCT* Home-based HIV Counselling and Testing; *HIV/AIDS* Human immunodeficiency virus and Acquired immune deficiency syndrome; *MSM* Men having sex with men; *PMTCT* Prevention of Mother to Child Transmission of HIV; *RCT* Randomized controlled trial*; PLWH* people living with HIV; *VCT* voluntary counselling and testing

**Table 2:** Coding tree for identifying several mechanisms and pathways for stigma reduction and HIV test uptake

Categories	Themes	Codes
Contextual factors	Structural factors	Homosexuality legislation, voluntary or mandatory reporting policies
	Health system factors	Health care quality and access, higher staff morale, effective referral, confidentiality, ongoing national health programs, discrimination at health care, increased partnership with community organizations
	Community factors	Social support, traditional beliefs and practices, gender norms, peer pressure, family testing, PLWH in the neighbourhood, gender-based violence, resource constraints, communication gap in the family (sexual taboo),
	Individual factors	Previous history of testing, gender, age, education, income, distance to health centre, urban-rural residence, increased risk-perception, self-confidence, higher self-esteem, intentions to test, trust in health care
Stigma Reduction Intervention Strategies	Awareness	Radio TV messages, mass media and interpersonal communication, film, health education program, role plays, group discussions, HIV advocate testimony, presentation, workshop, questions and answers, mobile phone messaging, training, motivational interviews, peer education
	Public health services	ART, opt-out testing, prevention from mother to child transmission, VCT, mobile VCT, home based VCT, Integrating HIV and RH services, community testing, family based testing and counselling, involvement of PLWH in the intervention
	Community mobilization	Child clubs, mobilization of community local counsellors, discussion with community leaders, mobilization of traditional health practitioners
	Support	Socio-economic support for community participation, contact with affected group, improving coping skills through involvement and empowerment, post-test support services, incentives
	Regulatory laws	Formation of hospital steering committee to oversee quality of care, hospital (confidentiality) policy development, material supply for practicing universal precautions, providing incentives for testing
Mechanisms of stigma reduction	Increase knowledge	Knowledge about HIV is manageable, prevention measures, changed negative beliefs, changed community norms, normalization, knowledge about universal precautions, changed sexual taboo
	Change attitude	Reduced fear (self-stigma), reduced perceived stigma (shame and worrisome), acceptance of testing services, increased tolerance and comfort with PLWH in variety of situations, acceptance of PLWH; respect for confidentially among health workers, less endorsement of policies to separate PLWH
	Change behaviour	Comfort, interaction of PLWH in the community, lower tendencies to exclude PLWH, less blame, reduced enacted stigma experiences, involving PLWH in the community, encouraging others to test
Outcome	Proximal outcomes	Improved knowledge, reduced fear, reduced shame, reduced blame, reduced discrimination and increased interaction
	Distal outcomes	HIV test uptake, self-efficacy and intentions to test for HIV

Note. *PLWH* people living with HIV; *VCT* voluntary counselling and testing

Identification and verification of the pathways followed an iterative process; connections were searched across data/themes to construct a cumulative picture. The generative mechanisms located within respective pathways were compared across similar and different contexts to identify if similar outcomes were generated and the preliminary programme theory could be improved. Finally, the preliminary programme theory was refined to reflect the generative mechanisms supported by evidence [22]. All interpretive processes were discussed and agreed upon among the co-authors of this review.

## Results

**Fig. 2:**
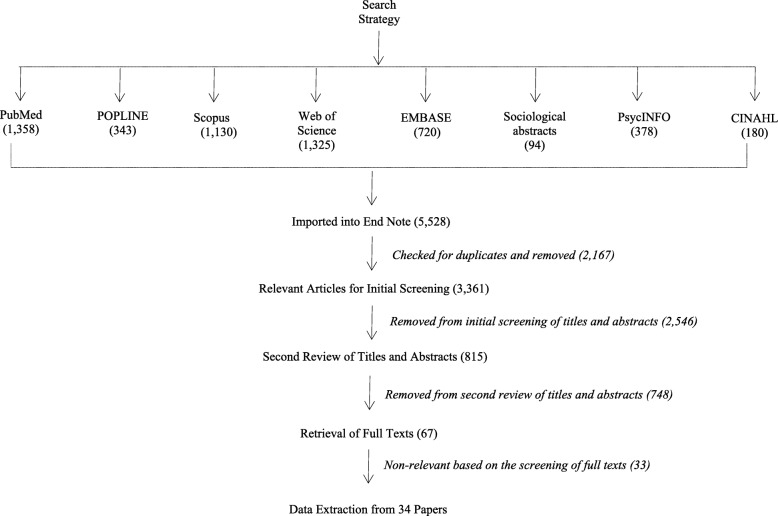
Systematic searching and selection of primary studies

Figure 2 shows the flow of work processes from the database selection to the screening processes and the final selection of primary studies. Searches for articles yielded 5528 records that were culled to 3361 records after removing duplicates. Abstracts were appraised for relevance against the inclusion criteria in the initial screening. A total of 815 articles were considered for a second screening. After the second screening, 67 articles were considered for a full text appraisal for relevance and resulted in 34 articles being retained for the synthesis.

### Study and intervention characteristics

Table 3 illustrates the study and intervention characteristics. From a total of 34 articles, 10 were cross-sectional studies, 8 were randomized controlled trials, 6 were qualitative, 5 were quasi-experimental, and 2 were a mixed-methods study (see Table 1). In addition, 1 article was a literature review and 2 were commentaries published in scientific journals. Regarding study quality based on the MMAT, approximately 68% of the articles were rated high quality. Two commentaries and one poster did not have clear research questions and methodological details, and therefore, were not assessed for study quality.

**Table 3:** Study and intervention characteristics

Characteristics	Number of studies	Percent (%)
Study types		
Randomized controlled trial	8	23.5
Quasi-experimental	5	14.7
Cross-sectional	10	29.4
Qualitative	6	17.7
Mixed-methods	2	5.9
Literature review	1	2.9
Commentaries	2	5.9
Study Quality		
100%	13	38.2
75%	10	29.4
50%	6	17.7
25%	2	5.9
Not applicable	3	8.8
Intervention characteristics		
Interventional studies	30	88.2
Non-intervention studies	4	11.8
Location of interventions (*n* = 30)		
African countries	18	60.0
South American countries	3	10.0
Asian countries	5	16.7
North America	1	3.3
Multi-country	3	10.0

There were 4 non-intervention studies [23–26] and 30 intervention studies. From a total of 30 intervention studies, most of the interventions were conducted in Africa (19), followed by Asia (5), South America (3), and North America (1). Three were multi-country interventions conducted in Lesotho, Malawi, South Africa, Tanzania, Zimbabwe and Thailand [27–29].

The proximal outcomes reported in all the intervention studies were: improved knowledge or changed attitude or changed discriminatory behaviour. The reported distal outcome was HIV testing uptake in 24 interventions studies and the remaining 6 intervention studies [30–35] measured either self-efficacy or intentions to test or both as distal outcomes. In these intervention studies, self-efficacy and intentions to test were conceptualized as proximal predictors of HIV test uptake. However, none of these studies suggested how self-efficacy and intentions lead to HIV testing uptake. Since our primary aim was to identify and verify the pathways and mechanisms of stigma reduction in terms of HIV test uptake, self-efficacy and intentions to test were not considered as outcomes in this review. HIV testing was mostly assessed by asking the study participants whether they had an HIV test within 12 months after the intervention. In the qualitative studies, participants were asked to explain their most recent HIV testing experience.

Among the 30 articles reporting interventions aimed at reducing stigma (in terms of attitudinal mechanisms), 15 interventions reported increases in HIV testing uptake, 8 reported reduction in stigma but did not report effectiveness in terms of HIV test uptake, 4 reported increases in self-efficacy or intentions to test and 3 reported interventions being ineffective to reduce stigma and increase HIV testing uptake.

### Stigma reduction intervention strategies

**Fig. 3:**
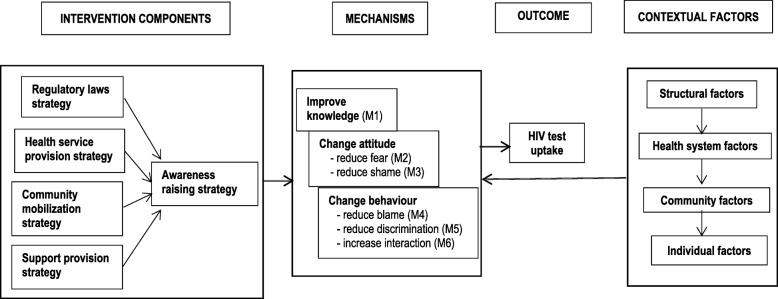
Programme theory illustrating mechanisms for stigma reduction in relation to the distal outcome of HIV test uptake

Our synthesis of all selected articles explained how stigma reduction interventions were implemented in relation to the opportunities or challenges provided by the contextual forces to increase HIV test uptake (see Fig. 3). The synthesis revealed five different intervention strategies that were included in the stigma reduction interventions, which are explained below:

#### Awareness raising strategy

The awareness raising strategy included HIV-specific fact-based written or verbal information, communication, and education as major components. This strategy was aimed at increasing knowledge, and changing attitude and behaviours. The awareness raising components in the interventions were: radio messages, printed mass media, interpersonal communication, films, health education programmes, role plays, group discussions, HIV advocate testimonies, presentations, workshops, questions and answers, mobile phone messaging, training, motivational interviews and counselling. All the interventions included in this review had the awareness raising strategy.

#### Health service provision strategy

The health service provision strategy included providing HIV-related services for the betterment of PLWH and the prevention of HIV infection in the community. This strategy reduced barriers related to health service access and logistics to increase HIV test uptake. The health services that were provided in the interventions were: anti-retroviral therapy (ART), routine health test without an informed consent (also called opt-out testing), availability of anti-retroviral drugs to mothers and their newborns, safe childbirth, infant feeding counselling, voluntary counselling and testing (VCT), mobile VCT, home-based VCT, family-based VCT, community VCT and other reproductive health services. Altogether, 18 interventions provided any of these health services, and 15 of them provided VCT services.

#### Community mobilization strategy

The community mobilization strategy involved local counsellors at the local community-level to enhance community organizing initiatives to raise awareness, change behaviours and provide health service to the community people. The local counsellors were traditional healers, youth counsellors, community health workers, local community leader or local facilitators. In the interventions, the mobilization of local counsellors was performed in the following activities: to organize community meetings on benefits of ART, to provide general information about HIV and HIV testing, to provide training to community leaders on ART and VCT, to work as a member of a community group that engages people in raising awareness of HIV, to perform HIV counselling and testing and to refer to the health clinic for standard testing and care. Altogether, 13 interventions had the community mobilizing strategy.

#### Regulatory law strategy

The regulatory law strategy included incorporating HIV-related legislation to protect and respect the human rights of PLWH. None of the interventions included in this review were based on regulatory law strategy. Only one intervention was based on developing policies related to confidentiality and respect of the rights of PLWH and formation of a hospital steering committee to oversee the quality of care. This intervention was implemented only in four hospitals of Vietnam, targeting the health workers [36].

#### Support provision strategy

The service provision strategy included providing social and psychological support to PLWH through teaching coping skills, directly contacting people associated with HIV and the friends or neighbours of PLWH and involving them in community development initiatives or health care and providing incentives or material support to the community people to access HIV-related services. Altogether, 7 interventions had the support provision strategy.

In total, 23 intervention studies had combined more than one strategy. Among the 23 combination interventions, 15 reported increases in knowledge and attitude leading to HIV testing uptake. A total of 8 combination interventions integrated awareness raising, health service provision and community mobilization strategies. Unlike the 23 combination interventions, the remaining 7 singular interventions had only the awareness raising strategy. From these 7 studies, 5 reported HIV testing uptake as an outcome, and 3 of the 5 studies reported increases in HIV test uptake via increasing knowledge and changing attitude [32, 33, 37].

### Mechanisms of stigma reduction in relation to HIV test uptake

**Fig. 4:**
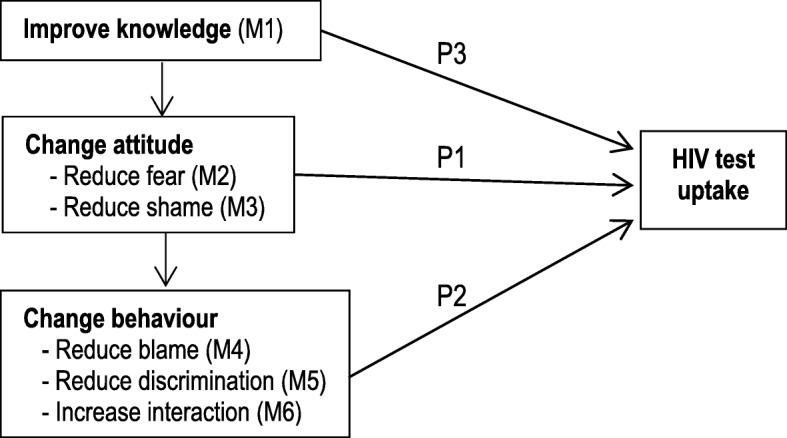
Pathways for stigma reduction in terms of HIV test uptake

Our synthesis of the evidence found six different mechanisms that were operating in stigma reduction interventions in relation to HIV test uptake, which were interlinked in three dominant pathways (See Figure 4). Pathway 1 and Pathway 2 illustrate the importance of knowledge in reducing stigmatizing attitudes and discriminations, whereas Pathway 3 illustrates the independent effect of knowledge on increasing HIV test uptake. These pathways were determined by the components of the interventions and the population group that were targeted by the stigma reduction interventions. Figure 4 only represents the pathways leading to increased HIV test uptake, and some inhibitory pathways are articulated narratively.

### Pathway 1: Knowledge leads to changes in stigmatizing attitudes to increase HIV test uptake

Pathway 1 indicates the pivotal role of knowledge (Mechanism 1; is abbreviated as ‘M1’) to trigger attitudinal mechanisms and increase HIV testing uptake. The attitudinal mechanisms triggered by knowledge were reduced fear (Mechanism 2; is abbreviated as ‘M2’) and reduced shame (Mechanism 3; is abbreviated as ‘M3’). Among the 22 intervention studies reporting increases in HIV test uptake, 11 of them [10, 27, 33, 35, 37–42] reported following Pathway 1, and 10 of 11 interventions were conducted in African countries (see Table 3). Interventions that followed Pathway 1 to increase HIV test uptake mostly had awareness raising as a singular strategy or had a combination of awareness raising and health service provision strategies and were targeted to the general population.

### Improved knowledge

Increases in knowledge were found to be the most common element to increase HIV test uptake in all the interventions. Altogether, 4 combination interventions (2 in African countries, 1 in multiple countries and 1 in Haiti) having awareness raising, community mobilizing and health service provision strategies were found to follow ‘Pathway 1’ to increase HIV test uptake [10, 27, 38, 41]. These interventions provided HIV testing and counselling services based on a ‘home-based door to door approach’ or a ‘whole community approach’, meaning that these interventions targeted all members of the community. This approach was noted to reduce the chances of being singling out from the mainstream health system among the vulnerable and hard-to-reach population groups and increased their participation in HIV testing services. In the study by White (2013), one of the participants from Cambodia mentioned [43]:

*“The local NGOs … visit once a month. They ask questions about my health*.. . *whether we are well or not, whether I take medicine regularly or not. I tell them that I take medicine every day. Home-based counselling and testing team also advises on the prevention of HIV transmission, including condom use”.*

Two interventions based on the awareness raising strategy noted a positive association between the programme exposure and knowledge, reduced stigma and increases in self-efficacy or intentions to test in Botswana and Malawi [30, 33]. Both interventions had multiple awareness raising components (e.g., radio-based activities, magazine-based information dissemination and distributing posters) and facilitated the formation of active community radio groups to increase awareness (M1). In addition*,* both interventions were complemented by national HIV testing programmes (Context; is abbreviated as ‘C’). In Africa, the HIV-related health care system (C), via increasing people’s access to health care and providing HIV prevention messages, has been independently changing community knowledge (M1) and attitudes (M2), and normalizing HIV testing uptake (Outcome; is abbreviated as ‘O’) [44].

### Reduced fear

Interventions promoting home-based testing and counselling in several African countries ensured confidentiality in relation to testing and keeping the HIV status a secret which reduced people’s fear of unwanted disclosure (M2) and led to an increased level of participation in HIV testing services (O) [10, 27, 41]. Contrastingly, in a community setting, most people might not consider having an HIV test because of fear associated with the lack of confidentiality [44]. Semugoma et al. (2012) described that, in a few African countries including Uganda, anti-homosexuality laws (C) had a negative effect on stigma reduction and HIV test uptake among men having sex with men (MSM) and the transgender population because of a perceived threat of disclosure of sexual behaviour and criminalization [26]. In addition, the mandatory reporting of a positive HIV test report policy (C) exacerbated the perceived threat of unwanted disclosure and discrimination and led to a lower uptake of HIV testing services among at-risk populations and the general population (O) [26].

Laws that promote mandatory reporting of HIV information (C) could lead to exacerbating fear of unwanted disclosure of HIV status. Working in South Africa, Weighs et al. reported that, in a workplace where HIV is stigmatized, an intervention that provided support via providing incentives for HIV testing was reported effective to increase knowledge, reduce fear and increase HIV testing uptake (Pathway 1) [45]. People who were tested due to the influence of an incentive were more likely to report a lower level of fear associated with HIV testing [37, 46]. These interventions could be more effective in a context where the laws and health care practices promote full rights of PLWH to maintain the confidentiality of all the information associated with HIV infection [47].

### Reduced shame

In South Africa, an intervention based on mobilizing local female counsellors ensured increased communication across different generations (M1), reduced shame (M3) for women to discuss sexual and HIV testing matters, and increased family support for those who tested positive [48]. Young girls and women in most African countries are generally ashamed to discuss sexual and HIV testing with parents and partners due to hierarchical relationships between generations, sexual taboos and intimate partner violence (C) [41]. In addition, these young girls and women would feel more comfortable talking to female counsellors, as it is regarded culturally inappropriate for men to counsel women in the absence of the husband [48]. In addition, Massey (2012) reported that African women who perceive greater risk for HIV infection were more likely to hold less stigmatizing attitudes and test for HIV [35].

### Pathway 2: Knowledge and attitude lead to changes in stigmatizing behaviour and consequently, increase HIV test uptake

Pathway 2 indicates the role of behavioural mechanisms to reduce discriminations among PLWH and consequently, increase HIV test uptake among at-risk or vulnerable populations. Seven interventions were found to follow Pathway 2 [28, 43–45, 48–50]. Three of the 7 were conducted in South Africa, 1 in Cambodia, 1 in Thailand, 1 in Mexico and 1 was a multi-country intervention (see Table 3). Two interventions conducted in South Africa aimed to mobilize local counsellors for counselling and HIV testing [44, 48]. Mobilizing local counsellors not only changed knowledge and attitudes but also the interventions triggered three behavioural mechanisms, namely, reduced blame (Mechanism 4; is abbreviated as ‘M4’), reduced discrimination (Mechanism 5; is abbreviated as M5) and increased interaction with PLWH (Mechanism 6; is abbreviated as M6).

### Reduced blame

Two interventions that mobilized traditional healers were effective in changing community misconceptions (M1), reducing the perceived threat of HIV (M2), reducing the tendencies to blame (M4) and excluding PLWH (M5) in South Africa. Consequently, these interventions increased HIV test uptake among at risk population groups and the general population (O) [44, 48].

Roura et al. (2009) and Moshabela et al. (2016) reported that, in some African communities, people believed that a wide array of diseases, including HIV, was caused by witches as a consequence of jealousy or revenge [40, 44]. Because of traditional beliefs and practices, most people living or associated with HIV consulted traditional healers instead of health care workers [44]. Traditional beliefs and practices (C) about disease aetiology lead to blaming PLWH, which was one of the reasons why people who are at risk of HIV were avoiding HIV testing. The traditional healers were often associated with church, religious and prophetic forms of healing and were considered influential figures of authority and had the power to persuade the community (C) [44].

A participant from Tanzania (2008) put it this way [40],

*In the past, the Sukuma called it “Kondela.” A person slims and looks like s/he is HIV positive, but s/he is not. It’s a disease similar to AIDS caused by medicines of the witches. It is often used if you quarrel with someone... if s/he has got that medicine s/he bewitches you.*

### Reduced discrimination

All four intervention studies that specifically targeted health care workers through awareness raising [29, 47, 50, 51] reported increases in knowledge; however, only three reported the interventions being effective to reduce the level of fear of transmission and discriminatory practices at health care facilities (e.g., reduced separating, labelling, marking in the records and giving nicknames; M5) and to increase health care workers’ motivation to provide services to PLWH [47, 50, 51]. Another intervention conducted in Thailand that involved PLWH as a health care provider led to reduced discrimination in health care (M5) and increased participation of vulnerable populations in HIV testing services (O). One of the participants from Thailand (2007) put it this way [49],

*“The project encouraged PLWH to become involved in their community. In the intervention village, two previously undisclosed PLWH joined the project and disclosed their status to other villagers and were welcomed into the community project.”*

By contrast, the anti-homosexuality legislation in Uganda punished health care providers and families of MSM if they offered help and support of any kind, including HIV testing. This legislation reinforced a homophobic environment in the health care system and broader community (C), and lead to fear and discrimination [26]. Based on the Ugandan experience, Semugoma (2012) noted that interventions aimed at reducing discrimination in a homophobic environment (C) are less likely to be effective in terms of HIV test uptake.

### Increased interaction with PLWH

Only two interventions implicated the mechanism of increased interaction with PLWH. For instance, in an intervention implemented in Thailand (2007), community leaders, youth volunteers and PLWH were involved together in identifying community needs, mobilizing community resources, generating additional income, and disseminating HIV/AIDS information in the community [49]. The intervention increased HIV knowledge (M1), increased interaction between PLWH (M6) and community people, and increased tolerance of PLWH in the community. Based on the experience from China, Wu et al. (2008) reported that engaging PLWH in delivering health care can also play a positive role to increase interactions between PLWH and the community people (M6), to increase knowledge about the situation of PLWH (M1) and to reduce discrimination in the community (M5) [47].

### Pathway 3: Knowledge leads to increases in HIV test uptake without changing stigmatizing attitudes and behaviours

Four intervention studies reported increases in HIV test uptake due to the influence of knowledge but without any concomitant changes in stigmatizing attitudes and behaviours [29, 32, 52, 53]. One of them was a film-based intervention based on the story of a heterosexual man in the context of Nigeria [32]. As the main male character in the film transmitted HIV to his fiancée via unprotected sex, this intervention had a positive effect on HIV testing uptake but a negative effect on changing the stigmatizing attitudes among Nigerian women [32]. A multi-country intervention study targeted PLWH, and the nurses reported increases in HIV test uptake without any reduction in the level of fear and perceived stigma [29].

In addition, one of these interventions was delivered in Kenya and reported changes in stigmatizing attitudes among community leaders (Pathway 2) but not among community members [52]. The increased HIV testing uptake among community members was due to a home-based testing approach without changing the stigmatizing attitudes. Based on the experience from China, Ma et al. (2008) reported that health care access also makes a difference in HIV test uptake, rather than other factors, such as stigma reduction [53]. It was also reported that some specific population groups, such as younger generations and people living in urban areas and having a higher income (C), were more likely to have better HIV knowledge and test for HIV.

## Discussion

Since the beginning of the epidemic, the majority of HIV prevention interventions have been based on awareness raising strategies [32]. Initially, these interventions only spread messages about HIV infection being linked with homosexual behaviour and drug use and defined HIV as an incurable, fatal and highly infectious disease. This message led to an increased level of perceived threat of HIV among the general population, which led to a negative evaluation of PLWH and created circumstances for exclusion and discrimination. On the other hand, there have been many achievements in the prevention and management of HIV, and the largest may be the development of highly active anti-retroviral therapy. Although HIV is no longer a fatal disease, HIV may not have been understood well by a considerable proportion of the population, and as a result, HIV stigma continues to persist in many communities.

Several behaviour-change models, such as the Knowledge-Attitude-Behaviour model and the Health Belief model, stipulate specific constructs and their relationships on how knowledge leads to changes in stigmatizing attitudes and behaviours [54]. These models, however, do not sufficiently consider contextual influences, which should still be described more comprehensively while addressing complex health behaviours, such as HIV test uptake. Our programme theory provides sufficient evidence to claim that knowledge is an essential component of stigma reduction. However, interventions aimed at increasing knowledge alone are insufficient to reduce stigma and lead to HIV test uptake.

Our review identifies some inhibiting pathways that influence the process of stigma reduction and HIV test uptake. The pathways, such as (a) anti-homosexuality laws leading to the creation of a homophobic environment at health care facilities, (b) traditional beliefs and practices about the disease aetiology leading to blame of PLWH, and (c) gender inequality that increases the feeling of shame among women while discussing HIV testing with their partner, increase stigmatizing attitudes and discourage people to test for HIV. Our review makes it clear why, how and for whom an awareness raising strategy should be complemented by other intervention strategies to change stigmatizing attitudes and behaviours, and consequently, increase HIV test uptake. Based on our programme theory, we propose the following points to be taken into account while designing and implementing an intervention to reduce stigma and increase HIV test uptake.

First, the interventions that are aimed at increasing HIV test uptake among at-risk or vulnerable populations should always take contextual factors into account. For instance, unless equal rights for MSM and the transgender population are ensured by law, stigma reduction interventions may not be effective to increase the participation of these specific population groups in HIV prevention. In this review, policies, such as anti-homosexuality laws and mandatory reporting of HIV test results, were found to create a homophobic environment with the health care system and broader community. Thus, policies, such as legal and social recognition of same-sex relationships or marriage and protection of sexual minority people from discrimination, should be implemented along with stigma reduction interventions to reduce stigma and increase HIV test uptake among the vulnerable and hard-to-reach population groups [55, 56].

Second, to increase the access of at-risk or vulnerable populations to HIV test uptake, it is important that the interventions followed Pathway 2 to trigger both attitudinal and behavioural mechanisms. Combination interventions having active community mobilizing strategies were more effective to reduce stigmatizing attitudes and behaviours and to increase HIV test uptake among at risk and vulnerable populations. The most consistently successful combination of strategies was awareness raising, health service provision and community mobilization strategies. Thus, our findings strongly support the concept of combination prevention, including biomedical (e.g., condoms, HIV testing), behavioural (e.g., awareness, counselling) and structural intervention strategies (e.g., decriminalization homosexuality, laws protecting PLWH), being more effective than individual prevention strategies [57].

Third, within the combination prevention approach, involving PLWH in providing health care was found to be promising to reduce discrimination. Involving PLWH in health care was found to have two major advantages. First, these PLWH directly benefitted from health care. Second, the networks initiated by these PLWH and vulnerable populations compared with the networks initiated by the HIV-negative general population were noted to be more effective to reduce fear, increase interaction of PLWH with other people and increase HIV test uptake [58]. Given that most of the developing countries are struggling to manage funds for the provision of routine HIV-related services, community mobilization strategies can still be implemented to increase awareness and encourage people to access health services for an effective and sustainable HIV/AIDS response in resource-limited settings [59].

Fourth, extending HIV testing uptake and coverage to underserved and hard-to-reach populations that do not necessarily access an existing health care service can be challenging. These populations are more likely to fear identification by being specifically targeted by HIV-related health services [38, 39]. Our review revealed that one way to increase the access of these population groups to HIV-related health services (e.g., HIV testing) was via targeting a wide range of individuals in the community. In addition, we also learned that HIV testing uptake was increased to a greater extent when testing was offered at a non-clinical setting, such as home-based testing, compared to testing offered at a clinical setting, because of easy access and perceived confidentiality.

Last, the difference in stigma experiences and HIV test uptake between communities could partly be explained due to the difference in socio-economic status and partly due to the difference in health care access [53]. Therefore, in a resource-limited setting, incentives can also be an effective way of encouraging and rewarding vulnerable people for a positive behaviour. Studies on incentivized HIV testing interventions in several LMICs have identified improved HIV testing uptake among vulnerable populations [60, 61]. In this review, we learned that incentives reduce the immediate logistical costs (e.g., transport and time) and the psychological costs (e.g., fear and stigma) in relation to HIV test uptake. Most importantly, in a resource-limited setting, the cost could be decreased by providing incentives as part of a lottery [45].

Stangl et al. (2013), in their review, have noted that the number, geography and complexity of stigma reduction interventions have expanded considerably, and, especially, a very high percentage of studies that showed reductions in stigma were of high quality [62]. However, they have also noted that most of these studies do not adequately address the manifestations of stigma on HIV test uptake. Our review also noted that data on interventions on stigma reduction and the influence of context on the intervention effectiveness to increase HIV test uptake are poorly documented. The data reported in some of the primary studies from where we had to pool insights were thin. Meaning that, the data were mostly descriptive, conceptually poor and had so little value for theorizing particularly on the context in which the interventions were rolled out. For instance, interventions based on regulatory law strategies are seldom implemented and tested for their impact on HIV test uptake, and limited literature currently exists on the link between structural factors and HIV test uptake. Therefore, in the future, researchers should be more motivated to consider why the stigma reduction intervention worked or did not work to increase HIV test uptake, and intervention studies on stigma reduction should pay more attention to the context to understand how the intervention worked.

### Limitations

A key limitation of this realist review is that we were very specific to include only studies with stigma reduction components and published in English. We also did not attempt to perform an independent search for national-level grey literature in the websites of Governmental and non-governmental organizations. As a result, our review may have missed uncovering some other contextual factors and mechanisms. For example, Figures 3 and 4 only depict the mechanisms leading to positive change and there are several inhibitory pathways that we have discussed in the narratives. Thus, we propose that the mechanisms outlined in this review should be seen as the key, rather than the only, mechanisms contributing to stigma reduction and HIV test uptake. Additionally, because of the qualitative nature of our review method, we were unable to define the cut-off of behaviour change level achieved to explain which particular interventions or the components of the intervention were more effective.

Despite these limitations, the use of a realist review is increasingly relevant in view of the contextualization of the issues, i.e., HIV-related stigma, implementation of interventions in different settings and contexts, as well as the psychological core constructs that render such interventions effective, in particular in LMICs. The scope of the review was broad, and not limited to one region (e.g., Sub-Saharan Africa), which added value in trying to achieve a saturation point in the number of ideas presented in the primary studies. There were 21 intervention studies that come from Sub-Saharan Africa and the studies that come from outside Sub-Saharan Africa also validated what have been learned from the studies conducted in Africa.

In addition, we included and synthesized all types of studies (i.e., quantitative, qualitative and mixed-methods) providing information about interventions, contexts, mechanisms and outcomes, surrounding stigma reduction and HIV test uptake. In this way, the review of evidence brought together a comprehensive perspective in terms of interventions and populations to the analysis and synthesis. In light of implementation research, there is a great need to better understand the general mechanisms that make stigma reduction interventions effective, and subsequently to collect evidence that goes beyond the typical experimental designs, which are not always feasible due to the complexity of interventions and the contexts in which they are implemented. The programme theory can be transferable to other communities and across diverse populations.

## Conclusion

The refined programme theory resulting from the realist review clarified why, how and under what circumstances interventions having singular or combination strategies are effective across different population groups. We found that these interventions produce an effect through two major pathways: (a) knowledge leads to changes in stigmatizing attitudes and increases in HIV test uptake, and (b) knowledge and attitude lead to changes in stigmatizing behaviours and consequently lead to HIV test uptake. In addition, this programme theory supports the adaptation of intervention strategies in terms of the socio-structural and cultural context and the population being targeted to reduce stigma and increase HIV test uptake in LMICs.

## Abbreviations

ART: Anti-retroviral treatment
CMO: Context-Mechanism-Outcome Configurations
HBCT: Home-based HIV Counselling and Testing;
HIV/AIDS: Human immunodeficiency virus and Acquired immune deficiency syndrome
M1: Mechanism 1
M2: Mechanism 2
M3: Mechanism 3
M4: Mechanism 4
M5: Mechanism 5
M6: Mechanism 6
MSM: Men having sex with men;
P1: Pathway 1
P2: Pathway 2
P3: Pathway 3
PLWH: People living with HIV
PMTCT: Prevention of Mother to Child Transmission of HIV
RCT: Randomized controlled trial
UNAIDS: Joint United Nations Program on HIV/AIDS
VCT: voluntary counselling and testing
WHO: World Health Organization
